# Integrative Use of Esketamine and Psychotherapy in a Real-World Outpatient Setting: A Mixed-Methods Case Series

**DOI:** 10.1192/j.eurpsy.2026.10921

**Published:** 2026-07-31

**Authors:** N. Ayad, S. Makhoul, F. alkhaja

**Affiliations:** Reem Neuroscience Center, Abu Dhabi, United Arab Emirates

## Abstract

**Introduction:**

Treatment-resistant depression remains a major clinical challenge, with many patients experiencing relapse despite advances in medication and psychotherapy. Esketamine offers rapid relief from depressive symptoms, while psychotherapy helps patients build lasting skills to manage their condition. Despite their individual effectiveness, little is known about how these treatments work together in real-world outpatient settings, particularly in non-Western populations.

**Objectives:**

This study seeks to contribute to this underexplored area by evaluating the combined use of esketamine and psychotherapy in a real-world outpatient clinic in the United Arab Emirates. By examining changes in symptom severity, as well as therapist and patient perspectives on treatment and relapse planning, the research aims to provide practical, culturally relevant insights into the integration of pharmacological and psychological interventions.

**Methods:**

Five adult patients receiving concurrent treatment were included. Quantitative data, such as depression and anxiety scores (PHQ-9, GAD-7) and treatment histories, were extracted from clinical records. Semi-structured interviews with patients and therapists explored experiences, treatment effectiveness, engagement, and relapse-prevention strategies. Interviews were transcribed and analyzed using reflexive thematic analysis.

**Results:**

Preliminary qualitative findings suggest that esketamine may increase emotional openness and cognitive flexibility, helping patients engage more fully in psychotherapy. Therapists reported improved responsiveness during sessions, while patients emphasized the role of psychotherapy in sustaining improvements beyond medication effects. Quantitative data analysis is ongoing and will be presented alongside qualitative insights.

**Conclusions:**

To our knowledge, this is the first study examining the integration of esketamine and psychotherapy in a Middle-Eastern clinical population. By combining clinical outcomes with patient and therapist perspectives, the study offers culturally relevant insights into real-world psychiatric care. These findings have important implications for relapse prevention, therapeutic alliance, and individualized treatment planning.

**Disclosure of Interest:**

None Declared

